# Metabolic Risk in Patients with a Diminished Ovarian Reserve and Premature Ovarian Insufficiency

**DOI:** 10.3390/jcm13175105

**Published:** 2024-08-28

**Authors:** Ralitsa Robeva, Atanaska Elenkova, Georgi Kirilov, Sabina Zacharieva

**Affiliations:** Department of Endocrinology, Faculty of Medicine, Medical University—Sofia, USHATE “Acad. Iv. Penchev”, 2, Zdrave Str., 1431 Sofia, Bulgaria

**Keywords:** diminished ovarian reserve, premature ovarian insufficiency, Turner syndrome, metabolic, hepatic, lipid, insulin resistance

## Abstract

**Objective:** Diminished ovarian reserve (DOR) and premature ovarian insufficiency (POI) represent conditions of different severity, characterized by an earlier-than-expected decrease in ovarian activity. The present study aims to compare metabolic disturbances between women with DOR and patients with POI from a different origin. **Materials and methods:** A total of 226 women (28 healthy women; 77 individuals with DOR, and 121 patients with POI/36 with Turner syndrome [TS] and 85 with non-TS POI/) have been studied retrospectively. Data concerning anthropometric indices, and metabolic parameters were collected. **Results:** Patients with DOR, non-TS POI, and TS had increased blood pressure and liver enzymes, pronounced insulin resistance, and worse lipid profiles than controls (*p* < 0.008 for all). TS patients had significantly higher ASAT, GGT, and TSH levels compared to non-TS POI and DOR individuals. The prevalence of type 2 diabetes tended to be higher in TS women compared to other groups. The prevalence of previously diagnosed polycystic ovarian syndrome was lower in the non-TS POI patients than in the DOR patients (*p* = 0.005). **Conclusions:** patients with decreased ovarian function suffer from insulin resistance, abnormal lipid profile, and subtle hepatic disturbances, irrespective of the severity of the condition and the presence of chromosomal aberrations.

## 1. Introduction

Premature ovarian insufficiency (POI) is characterized by decline in ovarian activity before the age of 40 years, leading to reproductive and endocrine disturbances [1,2]. Numerous genetic and non-genetic causes for POI have been described, such as chromosome X defects, genetic syndromes, monogenic variations, enzyme defects, autoimmune disturbances, iatrogenic effects, toxin exposure, and infectious diseases [3,4]. Nevertheless, no specific reason could be identified in most cases, with POI thus being considered an idiopathic condition [1].

Diminished ovarian reserve (DOR) represents a decrease in the quality and quantity of oocytes, leading to reduced fecundity in some women compared to their peers [5]. Unlike POI, DOR is a concept without a standard definition or widely accepted cut-offs of specific markers [6,7]. Nevertheless, DOR and POI have often been perceived as associated conditions in the common phenotypical continuum of premature ovarian senescence, varying in severity [8]. Early ovarian follicular exhaustion might result from a prenatally determined low-number ovarian reserve or accelerated follicular destruction during the postnatal period [9,10]. Both mechanisms might play a role in the development of DOR and POI, and the etiological causes for their development often overlap [9].

DOR and POI have been widely studied in the context of infertility and assisted-reproductive technologies [6]. However, their influence may extend far beyond reproduction, considering the substantial impact of hypoestrogenism on overall health and well-being [11]. Increased prevalence of metabolic disorders has long been described in patients with chromosome X abnormalities [12]. Patients with POI show a worse metabolic profile than healthy women [13]. Additionally, the presence of DOR has been related to lipid abnormalities and changes in granulosa cell cholesterol metabolism in infertile patients [14,15].

However, studies in distinct ethnic groups focused on lipid, glucose, and hepatic disturbances developing within the POI continuum are still insufficient. Thus, the present study aims to explore the differences between metabolic parameters in patients with DOR and POI due to different causes.

## 2. Methods

### 2.1. Participants

Medical files of all adult Caucasian women who had been consulted consecutively in a single tertiary Endocrine Department between 2006 and 2022 because of ovarian dysfunction [ICD-10 codes: E28.3, E28.8, E28.9, Q96] were analyzed retrospectively. All adult patients younger than 40 years, with increased FSH levels for their age (≥10 IU/L) were included in the study. Older women and patients with fluctuating FSH levels were excluded from the group. The complaints of the selected patients (*n* = 198) included menstrual disturbances, recurrent ovarian cysts, infertility, and unspecific perimenopausal symptoms (hot flashes, sweeting, palpitations, vertigo, insomnia, memory disturbances, joint pain, weight gain, tiredness, hair loss, increased hair growth, anxiety, and depressive mood).

A total of 121 women with premature ovarian insufficiency (POI) were selected. Thirty-six of them were with chromosomal aberrations (Turner syndrome/TS/group), demonstrated by karyotyping, while 85 women presented with non-chromosomal (non-TS) POI according to ESHRE criteria (oligomenorrhea and increased FSH levels ≥ 25 IU/L on two occasions, at least four weeks apart) [1]. In the case of several consultations, the results from the first admission were included in the study. Autoimmune ovarian failure was suspected in the individuals with at least one concomitant autoimmune disorder. Additionally, another 77 patients were selected based on increased FSH levels for their age (≥10 IU/L but <25) by at least one measurement, and were included as a group with diminished ovarian reserve (DOR group) [16]. The data of 28 consecutively recruited healthy female volunteers of reproductive age with regular menstruation, no hirsutism, normal prolactin, LH and FSH levels (<10 IU/L) and in euthyroid state were included as a control group.

### 2.2. Study Protocol

The Department of Endocrinology followed predefined algorithms in taking patients’ data, including family history, smoking, reproductive history, laboratory investigations, concomitant diseases, and therapy. The self-reported reproductive history included age of menarche, menstrual abnormalities (primary amenorrhea, secondary amenorrhea, oligomenorrhea, other menstrual disturbances, e.g., dysfunctional uterine bleeding), the number of pregnancies, and infertility (inability to conceive after 12 months or more of regular attempts). Laboratory parameters of women evaluated for ovarian dysfunction [ICD-10 codes: E28.3, E28.8, E28.9, Q96] have been taken in the early follicular phase of the menstrual cycle or in amenorrhea.

Information about anthropometric characteristics, laboratory parameters, gynecological history, ovarian diseases including polycystic ovarian syndrome (PCOS), operations and radiotherapy, as well as hormone-replacement therapy (HRT) of the patients were collected from the medical files and electronic medical records. In addition, the presence of arterial hypertension and type 2 diabetes was also registered.

The routine biochemical and hormonal investigations included follicle-stimulating hormone (FSH), fasting glucose (values available in 212 of 226 patients), total cholesterol (185/226), low-density cholesterol (LDL-ch) (161/226), high-density cholesterol (HDL-ch) (163/226), triglycerides (TG) (183/226), uric acid (107/226), creatinine (204/226), alanine aminotransferase (ALAT) (168/226), aspartate aminotransferase (ASAT) (168/226), gamma-glutamyl transferase (GGT) (127/226), fasting insulin (114/226), total testosterone (141/226) and thyroid-stimulating hormone (TSH) (206/226). These parameters were determined enzymatically by an automatic analyzer (Cobas Mira Plus; Hoffmann La Roche), while hormonal investigations were made through commercially available IRMA and RIA kits [17].

Unfortunately, specific genetic investigations aside from karyotype, e.g., *FMR1* gene premutation, had not been carried out routinely at the Endocrinology Department during the abovementioned period. Additionally, routine measurement of anti-Mullerian hormone (AMH), anti-ovarian or anti-adrenal antibodies was not provided. Thus, such data could not be extracted from the medical records.

### 2.3. Statistics

A total of 226 women were included in the final analyses. The results were presented as frequency (%) for dichotomous variables or as a mean [median] and standard deviation (SD) for continuous variables. Chi-square and Fisher’s exact test were used for the analysis of categorical data. The Kolmogorov–Smirnov test for normality of distribution showed a non-parametric distribution of most parameters. Therefore, non-parametric tests were used to establish differences between groups. Differences between the two groups were established through the Mann–Witney test, while multiple comparisons were calculated through the Kruskal–Wallis test. The Bonferroni adjustment for multiple testing was applied and the significance of the *p* value was set at 0.008 (0.05/6, considering comparisons between the groups). Statistical analysis was performed with MedCalc^®^ Statistical Software version 22.021 (MedCalc Software Ltd., Ostend, Belgium; https://www.medcalc.org; accessed on 15 May 2024).

## 3. Results

**Figure 1:**
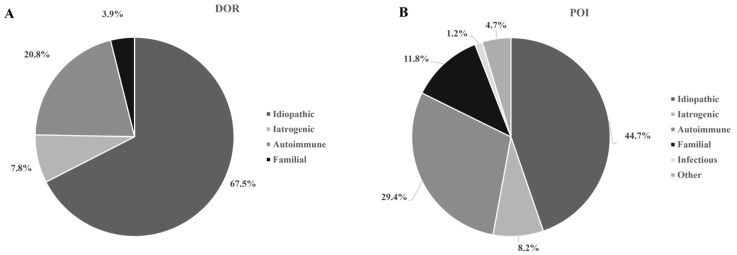
Etiology of diminished ovarian reserve (DOR) (**A**) and non-chromosomal premature ovarian failure (POI) (**B**). Patients with DOR showed slight differences in the etiology compared to non-chromosomal POI, with fewer autoimmune disorders and familial cases (*p* = 0.027). Iatrogenic causes included pelvic surgery, chemotherapy and/or radiotherapy for neoplastic disorders. Autoimmune origin was suspected in the case of at least one concomitant autoimmune condition.

The etiology of the DOR and non-chromosomal POI in the investigated women is presented on Figure 1. The main characteristics of participants with DOR, non-chromosomal POI and TS are presented in Table 1. Patients with TS were younger, shorter, and tended to be more obese than the other women, as expected, while other patients and healthy women were of a similar age and without differences in BMI. TS patients were former or current smokers three-times less often than the non-chromosomal POI patients. None of the healthy controls received hormone-replacement therapy (HRT), while 2.6% of women with DOR, 17.6% of POI patients and 50% of TS women were on HRT (*p* < 0.001).

None of the controls or DOR women, 15.3% (13/85) of non-chromosomal POI individuals and 69.4% (25/36) of TS patients presented with primary amenorrhea (*p* < 0.001). None of the included TS patients shared information about pregnancy attempts. A similar percentage of women with DOR and non-chromosomal POI had at least one pregnancy (37.7% vs. 36.5%, *p* = 1.000). In all but two patients, the pregnancies had occurred before the DOR/POI diagnosis. The self-reported prevalence of infertility was 18.2% among DOR women and 15.3% in patients with non-chromosomal POI (*p* = 0.676). No significant differences in the prevalence of reported non-specific perimenopausal symptoms were found between the same two groups (24.7% vs. 32.9%, *p* = 0.299). In TS and non-chromosomal POI patients with secondary amenorrhea, the menarche had occurred significantly later than in women with normal ovarian reserve or DOR (Table 1).

In patients with DOR, POI and TS, blood pressure levels were increased compared to healthy women (Table 1). The prevalence of hypertension was 6.5% in women with DOR, 10.6% in patients with non-chromosomal POI and 13.9% in TS women, while all controls had a normal blood pressure (*p* = 0.181). Diabetes mellitus type 2 had not been found in healthy women and individuals with DOR, while 2.4% of non-chromosomal POI and 8.6% of younger TS patients were diabetic (*p* = 0.033). Autoimmune thyroid disorder was more common in TS patients than in other investigated women, and they had increased TSH levels compared to other groups (Table 1).

None of the patients with TS reported the presence of polycystic ovarian syndrome (PCOS). On the contrary, 14.3% (11/77) of patients with DOR and 3.5% (3/85) of patients with non-chromosomal POI had been previously diagnosed with PCOS (*p* = 0.005). The prevalence of pelvic surgery interventions did not differ between the patients diagnosed with PCOS and other patients (*p* = 0.287).

**Figure 2:**
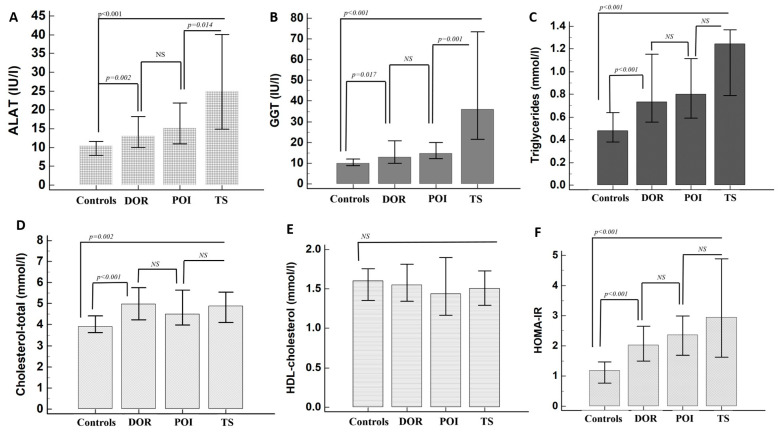
Hepatic and metabolic parameters ((**A**)—ALAT; (**B**)—GGT; (**C**)—Triglycerides; (**D**)—Total cholesterol, (**E**)—HDL-cholesterol, (**F**)—HOMA-IR) in the investigated women who had not been treated with hormone-replacement therapy. DOR-diminished ovarian reserve; POI–premature ovarian insufficiency (non-chromosomal); TS—Turner syndrome; NS—non significant.

The metabolic characteristics of patients are shown in Table 2. Patients with DOR, non-TS POI and TS had increased liver enzymes, pronounced insulin resistance and a worse lipid profile compared to controls. Patients with DOR tended to have increased LDL (*p* = 0.035) but lower ASAT (*p* < 0.001) levels compared to non-TS POI women, though other laboratory parameters were similar. TS patients were younger (*p* < 0.001), and with higher ASAT and GGT levels compared to non-TS POI and DOR patients. Similar results were obtained after the comparison of patients who had not been treated with hormone-replacement therapy (Figure 2). Similar results were obtained also when only idiopathic cases of POI and DOR had been considered.

Women with chromosomal and non-chromosomal POI on HRT showed lower FSH levels (32.3 vs. 72.3, *p* < 0.001), increased HDL-cholesterol levels (1.82 vs. 1.48, *p* = 0.026), and lower HOMA-IR (1.63 vs. 2.49, *p* = 0.026) compared to non-treated POI patients, while no differences in age, BMI and other metabolic characteristics were observed (*p* > 0.05 for all).

## 4. Discussion

Our data showed that the etiology of the DOR and non-chromosomal POI overlapped, as expected. However, familial predisposition was found more often in non-chromosomal POI patients than in women with DOR. Interestingly, approximately 14% of women with DOR had been previously diagnosed with PCOS, while the same syndrome was rare in POI patients. DOR might be seen in 17–21% of women with PCOS, despite the increased ovarian reserve in their early reproductive age, because of the exaggerated follicular decline [18,19,20]. However, complete ovarian decline is less likely in PCOS, considering the low prevalence of the syndrome among POI patients.

According to our results, DOR and POI patients showed increased insulin resistance, higher blood pressure, and worse lipid profiles than healthy women with normal FSH levels. Fasting glucose and insulin levels were similarly increased in women under 40 years old with DOR and POI, but the prevalence of overt type 2 diabetes was very low in both groups. Conversely, patients with TS tended to be more obese than other POI patients and showed higher type 2 diabetes prevalence. Different studies have shown an increased prevalence of obesity and carbohydrate disturbances in patients with TS compared to healthy women, progressing with age [21,22,23]. The specific susceptibility to type 2 diabetes in TS might be associated with impaired incretin effect, abnormal glucagon and growth hormone secretion, and intrinsic beta cell abnormalities [24]. Estrogen concentrations might not be the main factor responsible for the increased insulin resistance in the investigated women, considering the normal estrogenism in DOR participants. Several studies have suggested associations between DOR and cardiovascular risk factors [14,25,26]. For instance, young infertile women with DOR have shown increased HOMA-IR, TG, and LDL levels compared to healthy women with other infertility causes [14]. Lipid abnormalities were often found in patients with DOR, POI, and TS, and, interestingly, our patients with DOR tended to have even higher total cholesterol and LDL-cholesterol levels than POI patients. These results are consistent with the conclusions of recent meta-analyses, which have shown increased total cholesterol, LDL-cholesterol, and triglycerides in patients with POI, compared to controls [27,28]. Additionally, we did not find any differences in HDL-cholesterol levels between the groups, as in most previous studies [27,28]. However, opposite results have also been obtained in the scientific literature [13,29,30]; thus, the topic remains contradictory.

The worsening of the lipid profile in DOR and POI patients might be associated with androgen increase provoked by gonadotropin elevation. However, we did not find significant differences in testosterone levels between the investigated groups, though the healthy participants were only non-hirsute women without PCOS. On the other hand, a recent study showed that genetic polymorphisms associated with POI might be related independently to abnormalities in the lipid profile [31]. Thus, a subset of women might carry specific genetic variants associated with dyslipidemia, which could also predispose to early ovarian follicular decline.

Data about the hepatic function of women with premature ovarian aging are still insufficient. Our results showed elevated liver enzymes in women with DOR and non-chromosomal POI, compared to healthy women. Hepatic parameters in TS patients were significantly increased compared to both healthy women and patients with POI, reflecting the increased liver morbidity typical for the syndrome [32]. Similar findings regarding TS were described by previous studies [33,34]. However, in the study of Koulouri et al., liver enzymes did not differ between healthy women and POI patients [34]. Hepatic impairment in TS has been attributed to estrogen deficiency, immune abnormalities, and alterations in hepatic microcirculation [35]. Estrogen decline favors the development and progression of non-alcoholic fatty liver disease in postmenopausal women because of decreased insulin sensitivity, mitochondrial dysfunction, increased inflammation, and fibrogenesis [36]. However, the subtle liver-enzyme changes in women with DOR suggest that ovarian dysfunction might be associated with hepatic abnormalities even before developing severe hypoestrogenism. Larger studies are needed to explore the specific associations between metabolism, liver function, and ovarian senescence.

Interestingly, the median age of menarche was significantly delayed in patients with TS and non-chromosomal POI, compared to the DOR and control groups. Conversely, amongst American patients with infertility, early (before 13 years), but not late, menarche was associated with an increased risk of earlier follicular depletion [37]. Ethnic differences might be suggested, considering the pronounced variations in the median age of menarche reported by TS studies from different countries [23,38].

The prevalence of autoimmune thyroid disorder (AITD) among controls was similar to that previously reported in the same population [39]. Still, it doubled in patients with DOR and tripled in the POI group. Moreover, half of the adult TS patients had AITD, as in other studies showing increased thyroid autoimmunity in TS [40,41,42]. These results supported the findings of Bakalov et al., who described a stepwise increase in the prevalence of Hashimoto thyroiditis, defined as spontaneous hypothyroidism from healthy women to women with non-chromosomal POI, and to patients with TS [43]. However, our data showed that even a subtle decrease in the ovarian follicular pool might be associated with autoimmune thyroid disturbances. A recent meta-analysis revealed significantly lower AMH in adult women with thyroid autoimmunity, compared to controls [44]. However, the largest study on the same topic, including almost 5000 women, did not reveal any significant associations between the presence of thyroid peroxidase antibodies on the one hand, and the diminished ovarian reserve defined through the anti-Mullerian hormone categories on the other hand [45]. Thus, the negative correlation between thyroid autoimmunity and the antral follicular count might be observed only in a subset of patients with decreased follicular pool. The underlying mechanisms might include antigen cross-reactivity between ovarian and thyroid antibodies or a common genetically determined predisposition to autoimmune diseases [44,45,46].

It should be emphasized that the present study has multiple limitations, such as the retrospective design and the lack of AMH measurement. Therefore, the patients could not have been stratified using POSEIDON criteria [47]. Additionally, the FSH level >10 IU/L has very limited reliability for DOR diagnosis, due to inter- and intra-cycle variability [48]. Moreover, only one FSH measurement was available for most women in the DOR and control group. Furthermore, other unreported factors, e.g., the different time to TS or POI diagnosis, different duration, type, and adherence to the hormonal therapy, might have influenced the metabolic characteristics of the patients. However, the study included a wide heterogeneous group of POI patients with various symptoms and a large group of TS patients who had been referred to a single tertiary Endocrine Department. Thus, it extends the findings beyond the usually studied populations of infertile DOR and POI patients.

In conclusion, our data suggest that patients with DOR and POI suffer from pronounced insulin resistance, abnormal lipid profile, and subtle hepatic disturbances, irrespective of the condition severity and the presence of chromosomal aberration. Despite similar metabolic abnormalities in DOR and POI patients, differences in the origin and some characteristics have also been observed. Thus, these results indirectly support the assumption that DOR is not always a progenitor of POI, and both abnormalities might have different etiologies [49]. The established metabolic disturbances in women with DOR and POI might result from diminished steroid and peptide ovarian production, subtle thyroid dysfunction, specific genetic variants, or environmental factors and habits simultaneously violating metabolic processes and follicular development. Irrespective of the underlying mechanisms and study limitations, the present research showed lipid, carbohydrate, and hepatic abnormalities in women with DOR and POI, who were consulted, in everyday clinical practice. Thus, the target screening for dyslipidemia, glucose abnormalities, and metabolic-associated fatty liver disease might be beneficial not only for Turner syndrome patients, as per current guidelines [50], but also for patients with non-syndromic POI and even for women with increased FSH levels for their age. Lifestyle changes, increased physical activity, better adherence to hormone replacement therapy, and multidisciplinary care seem like simple coping strategies for metabolic improvement. However, only the long-term follow-up of patients with DOR and POI might estimate their true potential in preventing cardiovascular and hepatic complications.

## Figures and Tables

**Table 1:** Main characteristics of healthy women and patients.

	Healthy Women *n* = 28	Women with DOR *n* = 77	Women with POI *n* = 85	Women with TS *n* = 36	p_1_	p_2_	p_3_	p_4_	p_5_	p_6_	p_7_
Age (years)	28.32 ± 4.38 [29.00]	30.22 ± 6.10 [31.00]	30.14 ± 6.59 [31.00]	24.44 ± 6.66 [23.00]	0.089	0.084	**0.003**	0.941	**<0.001**	**<0.001**	**<0.001**
Height (cm)	167.32 ± 7.02 [168.00]	166.41 ± 5.79 [165.00]	163.89 ± 7.39 [164.50]	148.72 ± 6.90 [148.50]	0.469	0.044	**<0.001**	0.060	**<0.001**	**<0.001**	**<0.001**
BMI (kg/m^2^)	21.95 ± 4.49 [20.83]	23.28 ± 5.49 [22.18]	22.70 ± 4.77 [21.62]	24.94 ± 5.35 [23.99]	0.250	0.386	**0.005**	0.808	0.054	0.023	0.047
Smoking *	21.4%	22.1%	29.4%	8.3%	1.00	0.473	0.163	0.370	0.111	0.017	0.090
Systolic BP (mmHg)	103.57 ± 10.08 [100.00]	111.56 ± 11.60 [110.00]	113.14 ± 15.56 [110.00]	117.64 ± 18.80 [117.50]	**0.002**	**<0.001**	**<0.001**	0.720	0.162	0.264	**0.001**
Diastolic BP (mmHg)	67.86 ± 7.13 [70.00]	74.50 ± 8.23 [72.50]	74.59 ± 11.48 [70.00]	74.29 ± 9.73 [70.00]	**0.001**	**0.004**	**0.006**	0.744	0.952	0.805	**0.008**
Menarche (years)	12.68 ± 1.22 [12.50]	12.67 ± 1.45 [13.00]	13.61 ± 1.66 [14.00]	14.91 ± 1.51 [14.00]	0.840	**0.005**	**<0.001**	**0.003**	**<0.001**	**<0.001**	**<0.001**
AITD	10.7%	20.8%	29.4%	52.8%	0.389	0.075	**<0.001**	0.277	**<0.001**	0.022	**<0.001**
TSH (µIU/mL)	2.21 ± 1.06 [2.00]	2.16 ± 1.06 [1.90]	2.53 ± 2.04 [2.10]	7.78 ± 17.92 [3.00]	0.941	0.657	**0.001**	0.530	**<0.001**	**<0.001**	**<0.001**
Testosterone (nmol/L)	1.28 ± 0.59 [1.05]	1.81 ± 1.02 [1.60]	2.01 ± 1.52 [1.60]	1.47 ± 0.76 [1.45]	0.020	0.025	0.517	0.947	0.320	0.393	0.084
LH (IU/L)	3.87 ± 1.91 [3.30]	6.61 ± 5.45 [5.21]	29.43 ± 17.98 [25.30]	19.51 ± 16.45 [16.45]	**0.005**	**<0.001**	**<0.001**	**<0.001**	**<0.001**	**0.005**	**<0.001**
FSH (IU/L)	6.61 ± 1.83 [6.90]	13.83 ± 4.59 [12.60]	72.73 ± 38.75 [69.15]	60.60 ± 39.37 [64.00]	**<0.001**	**<0.001**	**<0.001**	**<0.001**	**<0.001**	0.104	**<0.001**

*—current or former smoker; BMI—body-mass index; BP—blood pressure; FSH—follicle-stimulating hormone; DOR—diminished ovarian reserve; POI—non-TS premature ovarian insufficiency; TS—Turner syndrome; AITD—autoimmune thyroid disease; p_1_—differences between healthy women and women with DOR; p_2_—differences between healthy women and women with POI; p_3_—differences between healthy women and women with TS; p_4_—differences between women with DOR and women with POI; p_5_—differences between women with DOR and women with TS; p_6_—differences between women with POI and women with TS; p_7_—differences among all groups. Bold—*p* ≤ 0.008.

**Table 2:** Main metabolic characteristics of healthy women and patients.

	Healthy Women *n* = 28	Women with DOR *n* = 77	Women with POI *n* = 85	Women with TS *n* = 36	p_1_	p_2_	p_3_	p_4_	p_5_	p_6_	p_7_
Glucose (mmol/L)	4.96 ± 0.37 [5.00]	5.12 ± 0.41 [5.17]	5.31 ± 1.09 [5.13]	5.30 ± 1.57 [5.10]	0.056	0.043	0.563	0.796	0.533	0.350	0.205
Insulin (µIU/mL)	6.10 ± 4.29 [5.05]	10.99 ± 7.64 [9.10]	11.58 ± 6.21 [9.60]	9.89 ± 5.83 [9.50]	**<0.001**	**<0.001**	0.018	0.271	0.878	0.366	**<0.001**
HOMA-IR	1.37 ± 1.06 [1.19]	2.47 ± 1.82 [2.02]	2.69 ± 1.56 [2.32]	2.15 ± 1.49 [1.74]	**<0.001**	**<0.001**	0.062	0.176	0.651	0.186	**<0.001**
Cholesterol (mmol/L)	4.16 ± 0.75 [3.92]	5.05 ± 0.99 [5.02]	4.84 ± 1.21 [4.50]	4.98 ± 1.11 [4.75]	**<0.001**	**0.006**	**<0.001**	0.171	0.550	0.506	**<0.001**
HDL-cholesterol (mmol/L)	1.58 ± 0.28 [1.60]	1.57 ± 0.42 [1.54]	1.64 ± 0.61 [1.48]	1.66 ± 0.39 [1.69]	0.717	0.707	0.403	0.987	0.329	0.421	0.768
LDL-cholesterol (mmol/L)	2.31 ± 0.61 [2.26]	2.99 ± 0.89 [2.94]	2.67 ± 1.03 [2.39]	2.84 ± 0.96 [2.66]	**<0.001**	0.192	0.020	0.035	0.500	0.354	0.009
Triglycerides (mmol/L)	0.60 ± 0.43 [0.48]	0.90 ± 0.48 [0.73]	0.99 ± 0.83 [0.79]	1.07 ± 0.79 [0.83]	**<0.001**	**<0.001**	**<0.001**	0.760	0.278	0.410	**<0.001**
ALAT (mmol/L)	11.06 ± 5.05 [10.45]	14.83 ± 6.25 [13.00]	19.19 ± 13.77 [15.50]	29.21 ± 28.21 [16.55]	**0.003**	**<0.001**	**<0.001**	0.174	0.013	0.155	**<0.001**
ASAT (mmol/L)	15.49 ± 3.92 [14.35]	15.88 ± 3.76 [15.40]	19.34 ± 6.02 [17.70]	27.25 ± 21.61 [19.00]	0.455	**<0.001**	**<0.001**	**<0.001**	**<0.001**	0.024	**<0.001**
GGT (mmol/L)	10.87 ± 3.75 [9.90]	16.33 ± 9.09 [13.00]	17.48 ± 9.91 [14.70]	57.34 ± 74.78 [27.20]	0.013	**<0.001**	**<0.001**	0.315	**0.004**	0.011	**<0.001**
Creatinine (mmol/L)	59.11 ± 7.45 [58.00]	57.83 ± 9.87 [56.00]	68.13 ± 99.58 [56.00]	51.16 ± 10.95 [49.00]	0.296	0.216	**0.002**	0.748	**0.004**	**0.007**	**0.007**
Uric acid (mmol/L)	261.22 ± 59.24 [265.00]	254.40 ± 73.66 [241.00]	273.47 ± 76.19 [262.00]	279.37 ± 71.15 [289.50]	0.731	0.706	0.482	0.343	0.192	0.625	0.631

DOR—diminished ovarian reserve; POI—non-TS premature ovarian insufficiency; TS—Turner syndrome; p_1_—differences between healthy women and women with DOR; p_2_—differences between healthy women and women with POI; p_3_—differences between healthy women and women with TS; p_4_—differences between women with DOR and women with POI; p_5_—differences between women with DOR and women with TS; p_6_—differences between women with POI and women with TS; p_7_—differences among all groups. Bold—*p* ≤ 0.008.

## Data Availability

Data are available from the corresponding author after reasonable request, and with permission from the local authorities.
